# Investigation of Shape with Patients Suffering from Unilateral Lymphoedema

**DOI:** 10.1007/s10439-017-1929-y

**Published:** 2017-09-20

**Authors:** Kevork Karakashian, Lawrence Shaban, Cheryl Pike, Raoul van Loon

**Affiliations:** 1 Zienkiewicz Centre for Computational Engineering, College of Engineering, Swansea University Bay Campus, Fabian Way, Swansea, SA1 8EN UK; 2 Lymphoedema Network Wales, Cimla Health & Social Care Centre, Abertawe Bro-Morgannwg University Health Board, Neath, SA11 3SU UK

**Keywords:** Edema, 3D camera, Swelling, Geometric analysis, Topology, Limb volume

## Abstract

This study investigates the use of a 3D depth sensing camera for analysing the shape of lymphoedematous arms, and seeks to identify suitable metrics for monitoring lymphoedema clinically. A fast, simple protocol was developed for scanning upper limb lymphoedema, after which a robust data pre- and post-processing framework was built that consistently and quickly identifies arm shape and volume. The framework was then tested on 24 patients with mild unilateral lymphoedema, who were also assessed using tape measurements. The scanning protocol developed led to scanning times of about 20–30 s. Shape related metrics such as circumference and circularity were used to distinguish between affected and healthy arms (*p* ≤ 0.05). Swelling maps were also derived to identify the distribution of oedema on arms. Topology and shape could be used to monitor or even diagnose lymphoedema using the provided framework. Such metrics provide more detailed information to a lymphoedema specialist than solely volume. Although tested on a small cohort, these results show promise for further research into better diagnostics of lymphoedema and for future adoption of the proposed methods across lymphoedema clinics.

## Introduction

Lymphoedema is a condition in which a patient suffers from chronic swelling of subcutaneous tissue as a result of deficient drainage of interstitial fluid.^11^,^16^,^17^ In homeostasis, an amount of fluid leaves the capillaries and flows into the interstitia. Under normal circumstances, most of this fluid is absorbed back into the blood stream and the lymphatic system pumps away the remaining imbalance in fluid. An interstitial fluid build-up occurs when the microvascular (capillaries and venules) filtration rate exceeds lymph drainage for a period of time; this is either due to a high filtration rate, low lymph flow or a combination of the two.^6^,^17^ A consequence of poor drainage is a rise of proteins and solutes in the soft tissue, which over time can cause fibrosis resulting in the soft tissue becoming harder and stiffer.^11^,^24^ The causes of an impeded lymphatic system can be divided into two main types, primary and secondary lymphoedema. Primary lymphoedema is characterised by impaired lymph vessels or lymph node development; this can be present from birth or develop throughout life.^24^ Secondary lymphoedema takes place as a consequence of damage to the lymphatic system due to cancer, trauma, infection, and obesity.^4^,^16^,^22^

Lymphoedema is currently treatable, but not curable. The condition can be treated through decongestive lymphatic therapy (DLT), which consists of manual lymphatic drainage (MLD), a specialised medical massage to soften and drain lymph fluid, compressive bandaging, decongestive exercises and skin hygiene procedures in the intensive phase.^9^,^22^ Furthermore, patient adherence to medical treatments as part of home maintenance approach plays a vital role in controlling the morbidity of lymphoedema.

The treatments mentioned above all have a direct influence on the topology/shape of the oedemic arm, which in return constitutes to change in volume. Thus, a common indicative measure of treatment efficacy is change in volume.^4^,^7^ Current methods of volume measurement consist of water displacement, circumferential limb measurements, perometry and bioimpedance.^11^ It should be noted that the latter method aims to measure fluid volume within the tissues, whilst the remaining methods are based on volume measurements of the entire limb. The water displacement method is based on the principle that the limb will displace its own volume in a 1:1 ratio with that of water, resulting in accurate measurements independent of geometrically complex shapes.^20^ For arms and legs circumferential limb measurements (CLM) are an alternative, which consists of subdividing the limb into regularly spaced segments and recording their respective circumference measurements using tape meters.^11^,^20^ Total volume is then the summation of all constituent segments *via* geometric formulae such as disc or frustum (truncated cone). The perometer is an optoelectronic device that relies on the occlusion of light to detect shape. Limb volume is then calculated by fitting an elliptical disc to the registered limb diameters.^2^,^23^ Lastly, an alternative method to monitor lymphoedema is bioimpedance, which measures the opposition/impedance to an alternating electrical current travelling through soft tissues.^3^,^19^ Both perometry and bioimpedance have shown to have high intra/inter-rater reliability values.^2^,^7^

Not all above assessment methods can provide geometrical data, with only CLM and perometer having the ability to provide circumference measurements. Even so, the use of such methods has been limited to calculate volume,^4^ without further investigation into shape. Therefore, the main objectives of this study were to:

- Examine the use of three-dimensional (3D) cameras to scan upper limbs and capture shape information.
- Identify different shape related metrics that could be used to monitor/diagnose lymphoedema.

Other studies that have used 3D cameras were merely interested in finding accurate volume measurements without analysing shape further in depth.^1^,^8^,^12^,^13^,^18^ Thus, due to the lack of information and tools available to analyse shape of oedemic limbs, a factor contributing to change in volume, we provide a cost-effective method and the tools necessary for creating metrics that could be used to monitor lymphoedema.

## Materials and Methods

**Figure 1:**
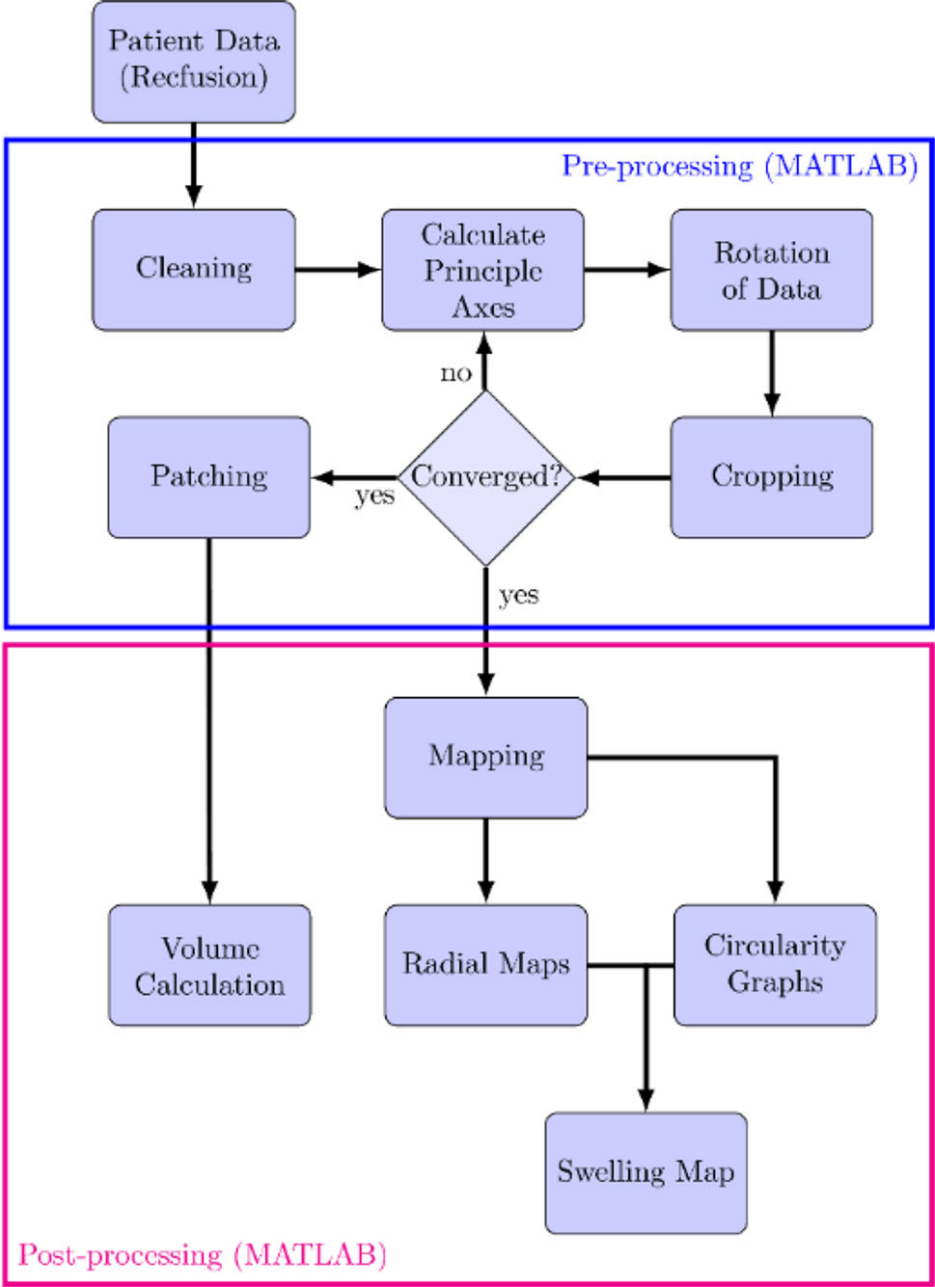
Flow chart of the pre- and post-processing framework starting from the 3D arm scans of patients. The “Pre-processing” and Radial Maps are discussed in the Appendix.

A framework (Fig. 1) was designed to systematically scan patients and process their data in an appropriate manner. This section discusses (1) patient inclusion criteria, (2) scanning protocol, and (3) post-processing of data for shape analysis, produced from the pre-processing section found in the Appendix. The pre-processing section involves cleaning unwanted objects from scans, rotation, cropping and patching of the scans.

### Patient Selection

For this study the patient group, consisting of 24 women aged between 29 and 76, was selected as part of a service evaluation of the lymphovenous anastomosis (LVA) programme. Patients eligible for this programme were to have mild unilateral lymphoedema. The inclusion criteria for mild lymphoedema was defined as: limb volume difference of less than 10%, normal limb shape, tissue is soft, tissues maybe pitting or non-pitting, no skin changes present, and no skinfolds or shape distortion present. Research and development approval for this service evaluation, which included the 3D scanning and analysis, was granted by the Abertawe Bro-Morgannwg University Health Board. The scanning was performed by the same lymphoedema specialist (Cheryl Pike, National MacMillan Innovation Lymphoedema Specialist) on two sites; Wrexham Maelor Hospital in Wrexham and Cimla Health and Social Care Centre in Neath. Patients’ arms, after their informed consent, were measured using 4 cm spaced tape measurements and scanned using a 3D camera. All scans used in this study were taken preoperatively.

### Scanning Protocol

Requirements for the scanning equipment and protocol were predominantly defined by the lymphoedema specialists in the clinic. The aim was to develop a fast and low cost alternative for assessing lymphoedema of the upper limbs. Therefore, a commercially available ASUS Xtion Pro 3D (A.X.Pro3D—ASUS, Taiwan) depth sensing camera was chosen in conjunction with software from RecFusion (ImFusion, Munich, Germany). The device consists of two electronic cameras that utilise the projection of structured light to capture 3D data. The first camera reproduces colour and brightness by detecting the reflection of visible light off the scanned surface. The second depth camera detects reflected infrared radiation to estimate distance. These two parameters are processed in RecFusion, resulting in a three-dimensional point cloud. This enabled us to capture triangulated 2D manifolds of the arm surface. These triangulations were then exported as STL-files (STereoLithography) containing the vertices and connectivity of the 3D points.

To ensure consistency in data capture, a scanning procedure was developed to control variables as well as possible. A stable camera rig was constructed from inexpensive off-the-shelf equipment (Fig. 9, Appendix A). The rig consisted of a camera tripod, ball joint mount, selfie stick with groove (to suppress rotation about its axis) and a 3D printed A.X.Pro3D camera mount in order to connect the camera to a standard 0.25 inch tripod mount. The total cost of rig, camera and software licence was just under 500 GBP. Where possible, the rig was chosen to utilize common camera equipment in order to aid clinical use by ensuring costs were kept to a minimum and replacement parts could be easily sourced. Patients were seated on a stool and asked to stretch out their arm horizontally ensuring the arm was at the same height as the ball joint by varying the height of the stool. The camera was then rotated 360° around the arm in about 20–30 s. Both arms of the patients were scanned in this manner.

### Post-processing

#### Volume Calculation

Volumes are used clinically to establish the severity of the swelling through comparison between affected and healthy limb (unilateral lymphoedema).^14^ Once a closed surface triangulation for the arm was established, the volume of the limb was calculated for comparison with tape measurement data. In current practice of CLM, the volume of an arm is calculated based on the assumption that arm cross-section is circular. First, the circumference of each cross-section was calculated as the perimeter from the structured grid mapped on the arm (Fig. 10b, Appendix B). Radius $R\left( z \right)$ was then derived from the calculated circumference, which was assumed to be $2\pi R\left( z \right)$. These radii were then used to calculate the cross-sectional areas $\pi \left( {R\left( z \right)} \right)^{2}$. Finally, the volume was calculated for each of the arm segments between 2 measurements as frustums and discs in Eqs. (1) and (2), respectively,

1 $$\begin{array}{*{20}c} {V_{\text{Frustum}} = \frac{\pi }{3}\mathop \sum \limits_{i}^{{N_{\text{m}} - 1}} \left( {R_{i}^{2} + R_{i + 1}^{2} + R_{i} R_{i + 1} } \right)\left( {z_{i + 1} - z_{i} } \right),} \\ \end{array}$$

2 $$\begin{array}{*{20}c} {V_{\text{Disc}} = \pi \mathop \sum \limits_{i}^{{N_{\text{m}} - 1}} \left( {\frac{{R_{i} + R_{i + 1} }}{2}} \right)^{2} \left( {z_{i + 1} - z_{i} } \right),} \\ \end{array}$$

where $N_{\text{m}}$ is the number of measurements taken and $R_{i} := R(z_{i} )$.

An alternative, more accurate, formulation for the volume was found through the divergence theorem, which states that in the absence of creation/destruction of mass, density of an object can only change through the flow of mass in or out of the boundaries. Hence, the total volume of the closed manifold was calculated as,

3 $$V = \int\!\!\!\!\!\int\!\!\!\!\!\int {{\mathbf{\nabla}} \cdot \user2{F}dV}=\mathop{{\int\!\!\!\!\!\int}\mkern-21mu \bigcirc} {\user2{F} \cdot \user2{n}dA}$$

where $\varvec{F}$ is a vector field, $\nabla\cdot$ is the divergence and $\varvec{n}$ the surface normal. Note that $\varvec{F} = [x,0,0]$ leads to $\nabla \cdot \varvec{F} = 1$, which is a valid solution to Eq. (1). The integral across the closed surface can now be evaluated discretely with vector $\varvec{x}$, the *x*-coordinates of the centroids of all surface triangles. If triangle $j$ is one of the triangles describing the triangulated arm surface, then the volume can be calculated as

4 $$\begin{array}{*{20}c} {V \approx \mathop \sum \limits_{j = 1}^{{N_{\text{f}} }} \mathop \sum \limits_{i = 1}^{{N_{\text{d}} }} F_{j,i} {n}_{j,i} A_{j} } \\ \end{array}$$

where $$N_{\text{f}}$$ is the number of vertices and $$N_{\text{d}}$$ is the number of dimensions. Equations (2) and (3) were subsequently used to calculate arm volumes using different segment lengths ($$z_{i + 1} - z_{i} )$$, and compare them with the more accurate integral method, Eq. (4).

#### Circularity

One of the known drawbacks of the tape measurements is that it relies on the idea that the arm is approximately circular. The 2D manifold allowed us to evaluate how true this assumption is and a measure of circularity could help with this. Hence, a relation for the dimensionless parameter circularity, *C*, was defined based on perimeter and cross-sectional area that has the value one for a perfectly circular cross-section and values less than one for distorted shapes.

5 $$\begin{array}{*{20}c} {C = \frac{{2\sqrt {\pi \;{\text{Cross - sectional }}\;{\text{area}}} }}{\text{Perimeter}} = \frac{{2\sqrt {\pi A} }}{P}} \\ \end{array} .$$

This value can also be used to identify non-circular anatomical features on the arm such as the elbow. Finding such a clear anatomical landmark in an automated manner was important for realignment of consecutive scans of the same arm in order to calculate volumes consistently.

#### Swelling Maps

When scanning the two individual arms for a given patient or the same arm at different points during treatment, there will always be variability in the orientation of the arm and inconsistency in the identification of the wrist (currently the only user input required). This hindered a straightforward comparison of scans and a 2-step process was introduced to make a consistent comparison possible. First, the elbow was identified using the circularity metric introduced earlier. This then allowed alignment in *z*-direction by imposing the same *z*-coordinate for the arm scans to be compared. The second step was a cross-correlation between the radial maps (discussed in the Appendix) of both scans, which relies on the fact that the shapes of the right and left arm have similar features. Both radial maps, *f* and *g*, were multiplied together after each successive circumferential shift of $\Delta \theta_{j} = 2\pi /N_{\theta }$. After every shift the cross-correlation $\sigma$ was calculated as,

6 $$\begin{array}{*{20}c} {\sigma = \mathop \sum \limits_{j = 1}^{{N_{\theta } }} f\left( {\theta_{j} ,z} \right)g\left( {\theta_{j} + \Delta \theta_{j} ,z} \right)}, \\ \end{array}$$

and the shift required to align arms circumferentially could then be defined by $\mathop {\arg \text{max} }\limits_{{\Delta \theta_{j} }} \sigma$. After the alignment, it was possible to subtract radial maps between the affected and healthy arm which resulted in a radial difference map indicating those areas on the arm that were swollen relative to the healthy arm. If a given arm is scanned at different points in time the radial differences will indicate in which areas swelling has gone down or where swelling has occurred. This could be a valuable monitoring tool for clinicians.

#### Data Clustering

To further examine the effect of shape, patients were sought to be grouped together using volume and circularity measurements. If these metrics gave similar patient groupings, it can be concluded that shape has no additional effect in distinguishing different classes of patients/lymphoedema with respect to volume. On the other hand, if different clusters were produced, then shape could be used as a complimentary metric to study lymphoedema, being more sensitive to changes in topology when comparing healthy and oedemic arms. Patient grouping was achieved *via* k-means clustering. In this iterative process, initial cluster centres are specified at random, assigning the closest surrounding observations/data points to each cluster centre. The cluster centres then move towards the mean position of surrounding observations till there is no change in data grouping. Since the position of oedema varies from one patient to another, k-means was applied on lower arm and upper arm data.

## Results

The data of 24 patients with mild lymphoedema was processed, analysed and compared using the methods described in the previous section.

### Reliability of Scanning and Post-processing

First a suitable scanning process is defined for the arms ensuring repeatability and speed. The same arm of a healthy subject was scanned 10 times over a period of 10 min, post-processed to output volumes starting from wrist to a height of 35 cm, leading to a mean and standard deviation (SD) of 2.5222 ± 0.0250 L. The coefficient of variation was 0.9911%. This suggested that the rotation speed and starting point of the camera have a very small influence on the final reconstruction of the arm. To analyse the effect of wrist identification, a second test was conducted by identifying the wrist five times for a given scan. This led to a mean volume of 2.5151 ± 0.0058 L, suggesting that the wrist can be determined consistently.

As the first two tests measured the reliability of the camera to detect the same shape/arm, the reliability to detect different shapes/arms was also assessed. Both arms of seven healthy subjects were scanned three times, and a test–retest analysis was carried out using intraclass correlation coefficient (ICC). Volume measurements were calculated based on a segment starting from the wrist with a height of 40 cm. ICC and its 95% confidence intervals were calculated using the statistical software SPSS (Table 1). The single form 2-way mixed model, ICC(3,1), was used along with the absolute agreement type. We choose not to generalise our ICC findings by using ICC(3,1) rather than the 2-way random model ICC(2,1), since the rater is fixed and the repeated arms are no longer considered to be random.^10^,^25^ Nonetheless, according to McGraw and Wong formulations,^15^ also used in SPSS, both ICC(2,1) and ICC(3,1) will yield equal results.

**Table 1:** ICC results for volume calculations based on single rating, absolute agreement and 2-way mixed model.

	Intraclass correlation	95% confidence interval	
		Lower bound	Upper bound
Single measure	0.957	0.898	0.985

Carrying out the same repeatability test using mean arm circumference measurements, rather than volume, resulted in a ICC(3,1) value of 0.946 with lower and upper bounds of 0.869 and 0.981 respectively.

The last factor considered was the resolution of the scan, which can be defined by the user in the RecFusion software. The resolution of scans was tested by completing three scans of the same limb taken at high (512 voxels at 1.95 mm), medium (256 voxels at 3.9 mm) and low (128 voxels at 7.8 mm) resolutions. The reduced resolutions effectively result in an STL mesh consisting of fewer but larger triangles. This evidently increases the volume of the STL as the lowest resolution has an increased volume of 5.76% when compared to that of the highest resolution (Table 2). Based on these results the high-resolution scans were used in the remainder of this work.

**Table 2:** Influence of scanning resolution on volume.

	Resolution		
	High	Medium	Low
Volume (L)	2.5072	2.5369	2.6516

### Volume Calculations

**Figure 2:**
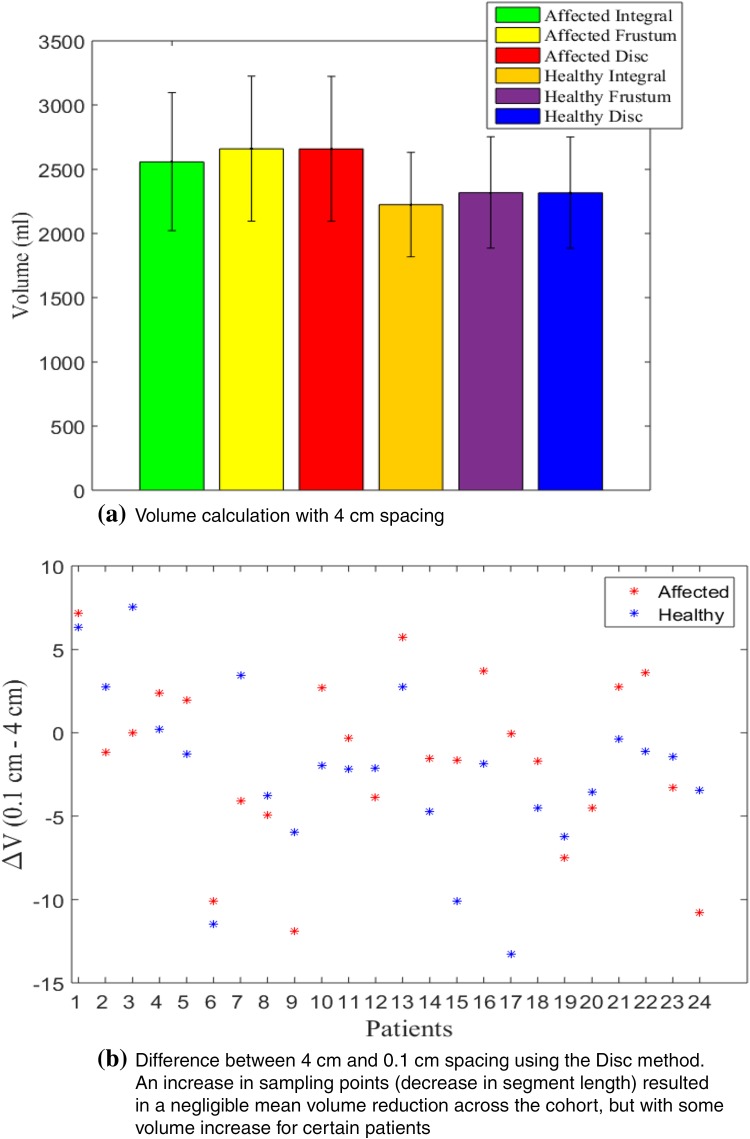
(a) Comparison between the three main volume calculations; integral, frustum and disc methods. Data for all 24 patients with mean arm volumes are indicated by bar height, and standard deviations with vertical lines; (b) comparison between the 4 and 0.1 cm intervals highlighted for disc method.

Volume is a standard metric used to establish the severity or monitor the progression of lymphoedema.^4^,^7^ The 3D arm scans allow the investigation of errors introduced when using approximations such as the frustum or disc method (Fig. 2a). The number of sampling points along the arm was increased resulting in spacings from 4 to 0.1 cm i.e., the height $\left( {z_{i + 1} - z_{i} } \right)$ in Eqs. (1) and (2) was equal to 4 and 0.1 cm respectively. As the spacing was decreased from 4 to 0.1 cm, average volume of all affected arms decreased by 2 and 1 mL using frustum and disc methods respectively, and a decrease of 4 and 2 mL was noticed for healthy arms respectively. It should be noted that this decrease is an average, as the 0.1 cm spacing increased limb volume for some patients (Fig. 2b).

**Figure 3:**
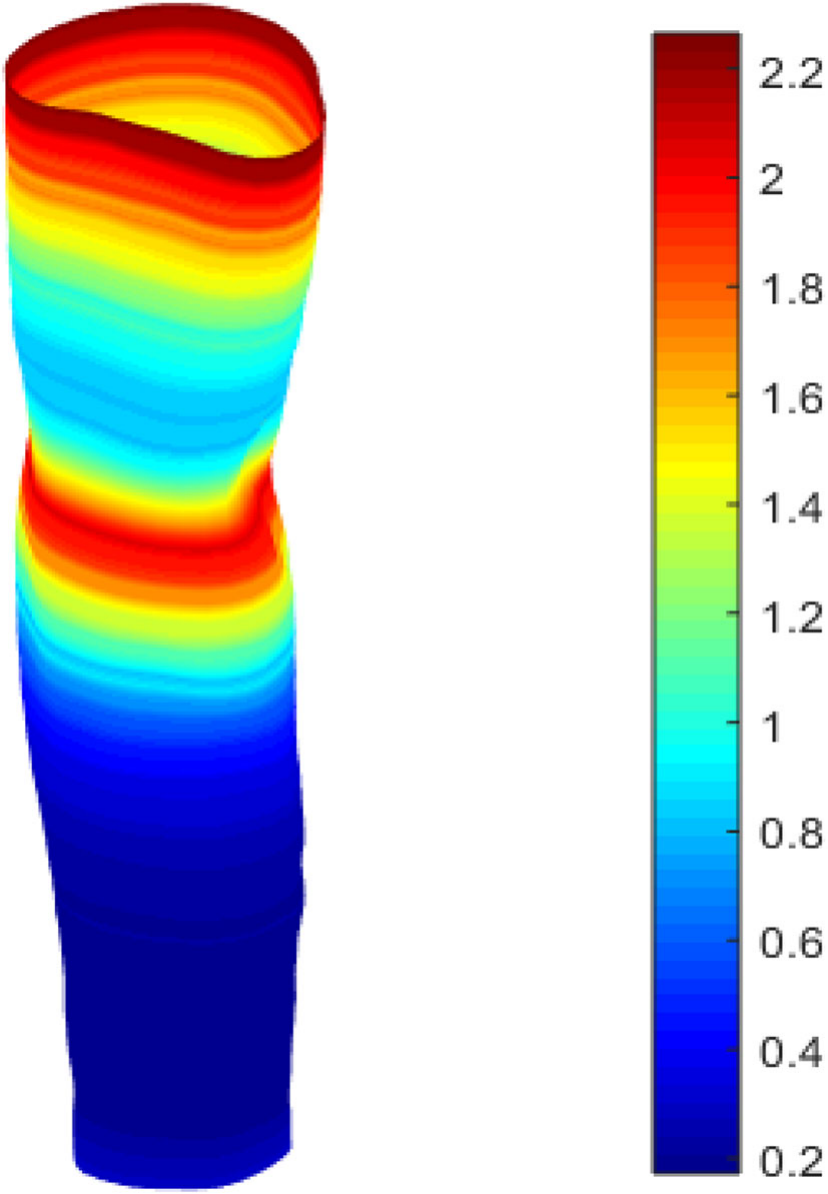
Volume error calculation of disc-minus-integral (mL). Large errors/differences between the disc and integral method are depicted by red. Due to the assumption of circularity, the mean error from all 24 patients was found to have a peak at the elbow, and another on the upper arm. This was true for both affected and healthy arms. The sampling interval was 1% of the normalised height ≈ 0.3 cm.

**Figure 4:**
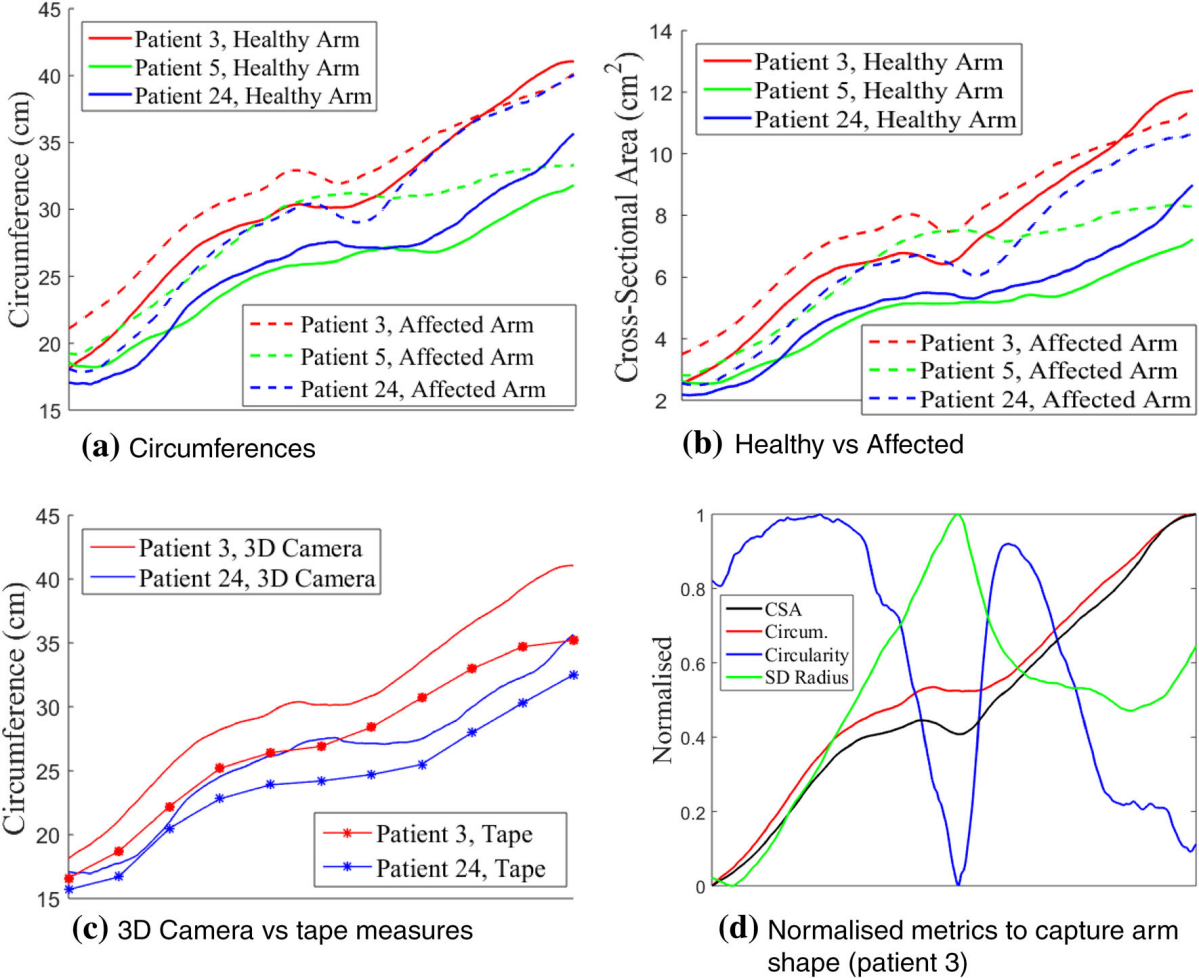
Geometric analysis of affected and healthy arms. The *x*-axis displays the axial direction along the arm in all graphs, starting from the wrist (left end of graphs), and ending at the armpit. Patients analysed were chosen randomly. (a) Circumference trend for three affected and healthy arms (3D camera); (b) comparison of healthy vs. affected (3D camera); (c) Comparison of healthy arms between 3D camera and 4 cm tape measures; (d) Normalised circumference, cross-section area, circularity and standard deviation of radius.

The volume difference between integral and the other circular methods was calculated to see which positions along the arm suffer most from the assumption of circularity. Not all scans were cropped at the armpit. To allow for a sensible comparison all arms were therefore normalised by the length of the lower arm. Hence, all arms started at the wrist and ended at the same anatomical position on the upper arm. Two positions of maximum error were noticed, one at elbow, having the least circular shape as shown in the next section (Fig. 4d), and the other on the upper arm, where the skin is most loose (Fig. 3).

### Circumference, Cross-Sections and Circularity

This section highlights the relevance of some geometric features of the arms and identifies statistically significant differences between affected and healthy arms.

The circumference of an arm is intuitively smallest at the wrist with a gradual incline towards the elbow. At that point the circumference experiences a short decline after which it gradually increases further. This trend is shown for the affected arms of three selected patients in Fig. 4a.

Figure 4b shows a comparison of the healthy arm vs. the affected arm. This illustrates the variability in circumference changes between patients as a result of the lymphoedema.

Figure 4c demonstrates the difference between the tape measurements and the 3D camera scans. It can be observed that the lower resolution sampling of the tape measurements results in a loss of detail compared to the scans. Shape features of the arm might be missed as a result of this. The difference between the camera and tape measures was typically larger near the elbow region, which will enhance any differences in the calculation of volume as described in the previous section. This could be due to the irregularities in circumference, which are significant in this area with concave and convex parts. The circularity of the arm can either be investigated by plotting the standard deviation of the radius or the circularity as defined in Eq. (5) along the axial position of the arm. In order to compare the shape of these curves with the circumference distribution, all curves were normalised and plotted in Fig. 4d. This shows that the circularity reduces as you move towards the elbow. At the elbow, there is a very clear minimum and maximum for both the circularity and standard deviation respectively. These are more pronounced than the dip in the circumference graphs and it can be clearly identified for all patients. Hence, circularity can be used as a clear distinguishable marker on the arm, which is useful when seeking an automated comparison between the healthy and affected limb or when identifying oedema progression when monitoring the patient over longer periods of time.

**Figure 5:**
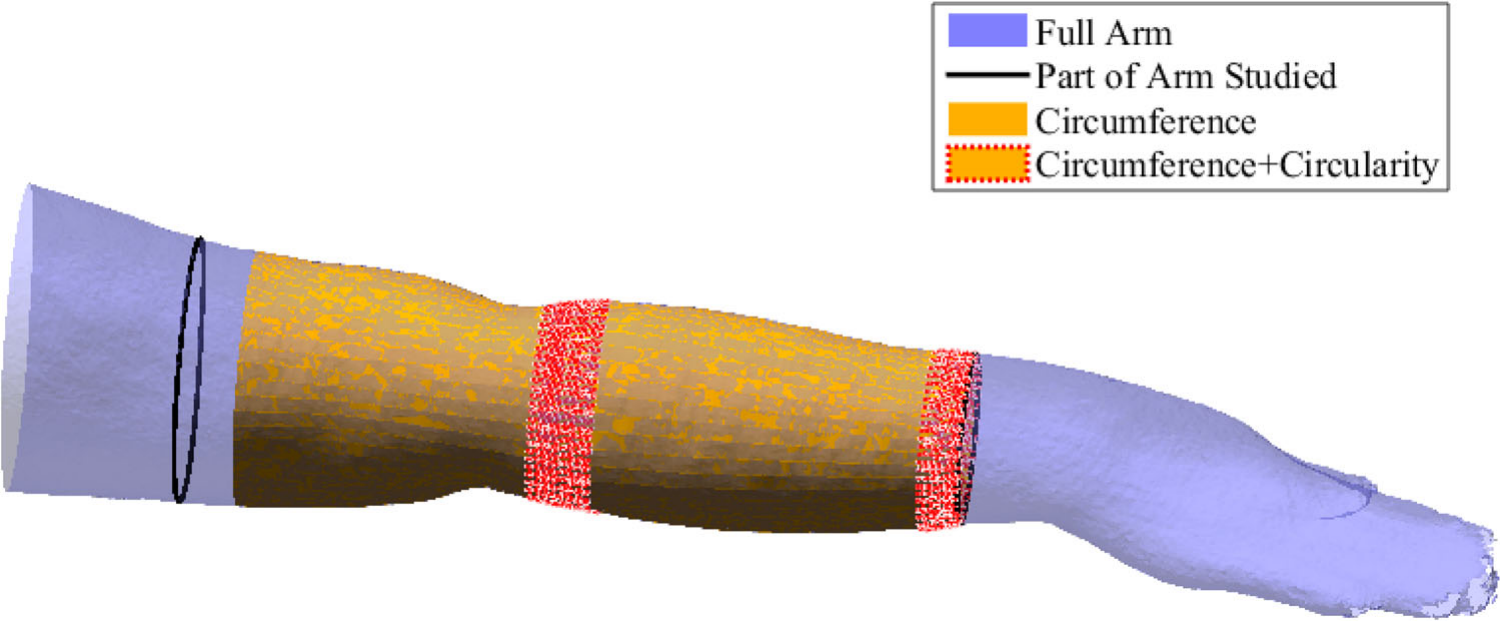
Results of paired-sample t-test between affected and healthy arms for all 24 patients visualised on a random arm. Regions shaded with orange are locations where circumference measurements from the 3D scans were able to distinguish affected arms from healthy. Circumference also shared locations with circularity measurements, shaded in orange and red, where such distinction was true. The whole scan is visualised in light blue. Region of study is bounded with dark lines. It encompasses a percentage from the whole arm around the elbow, due to different arm lengths as discussed in “Circumference, Cross-Sections and Circularity”.

Paired-sample t-test was carried out to distinguish healthy and affected arms using circumference and circularity measurements. To compare the same anatomical regions across all 24 patients, and due to the variability between forearm/upper lengths of patients, the elbow was chosen as a reference point, from which a proportion of the total arm length was added or subtracted to study the region above and below the elbow. Healthy and affected forearms can be distinguished using circularity, whilst both the upper and lower arms could be identified using circumference (*p* ≤ 0.05). The positions across which these statistical differences occurred are visualised on a random arm in Fig. 5. When the healthy arms of all 24 patients were divided into two groups, the t-test was incapable to distinguish between them (*p* ≤ 0.05).

### Data Clustering

**Figure 6:**
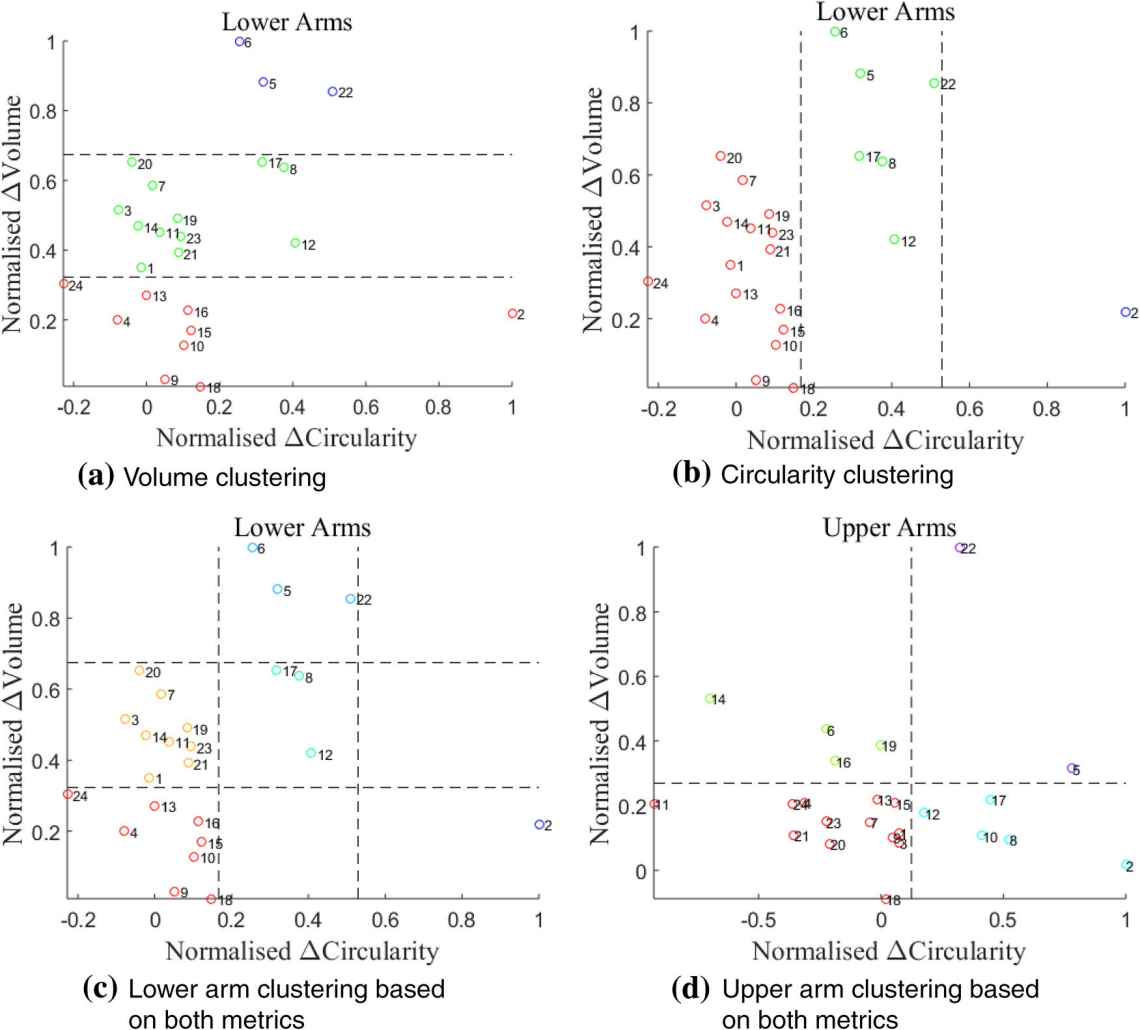
K-means clustering applied on lower and upper arms of 24 patients. Different clusters are separated by colour and dashed lines. Patient numbers are written next to the scatterplots. (a, b) show different groupings based on the choice of metric. Combining both metrics results in a 2D cluster, with different patient groupings for lower and upper arms respectively.

As mentioned earlier, the difference between the affected and healthy arm is a good indicator for monitoring unilateral lymphoedema. Therefore, k-means was applied using the metrics ${\text{Volume}}_{\text{affected - healthy}}$ and ${\text{Circularity}}_{\text{affected - healthy}}$. Figure 6 shows different patient groupings based on volume, circularity and the combination of both. Lower arms resulted in different patient grouping when compared to upper arms. The results clearly indicate different groupings of patients, which could be used to correlate patient outcome with the initial state of the arm.

### Oedema Maps

In the previous section, it is shown that there is variability in the arm shape between different patients. However, the aim is to develop a framework that would be able to distinguish patient groups and target their treatment accordingly. Metrics such as circularity or gradients in circularity could be used, but even those metrics would “hide” topological information of the arm. Therefore, a more detailed study of the geometry is proposed in this section where the entire arm surface is considered alongside the contour maps as described in the post-processing section of the Appendix.

**Figure 7:**
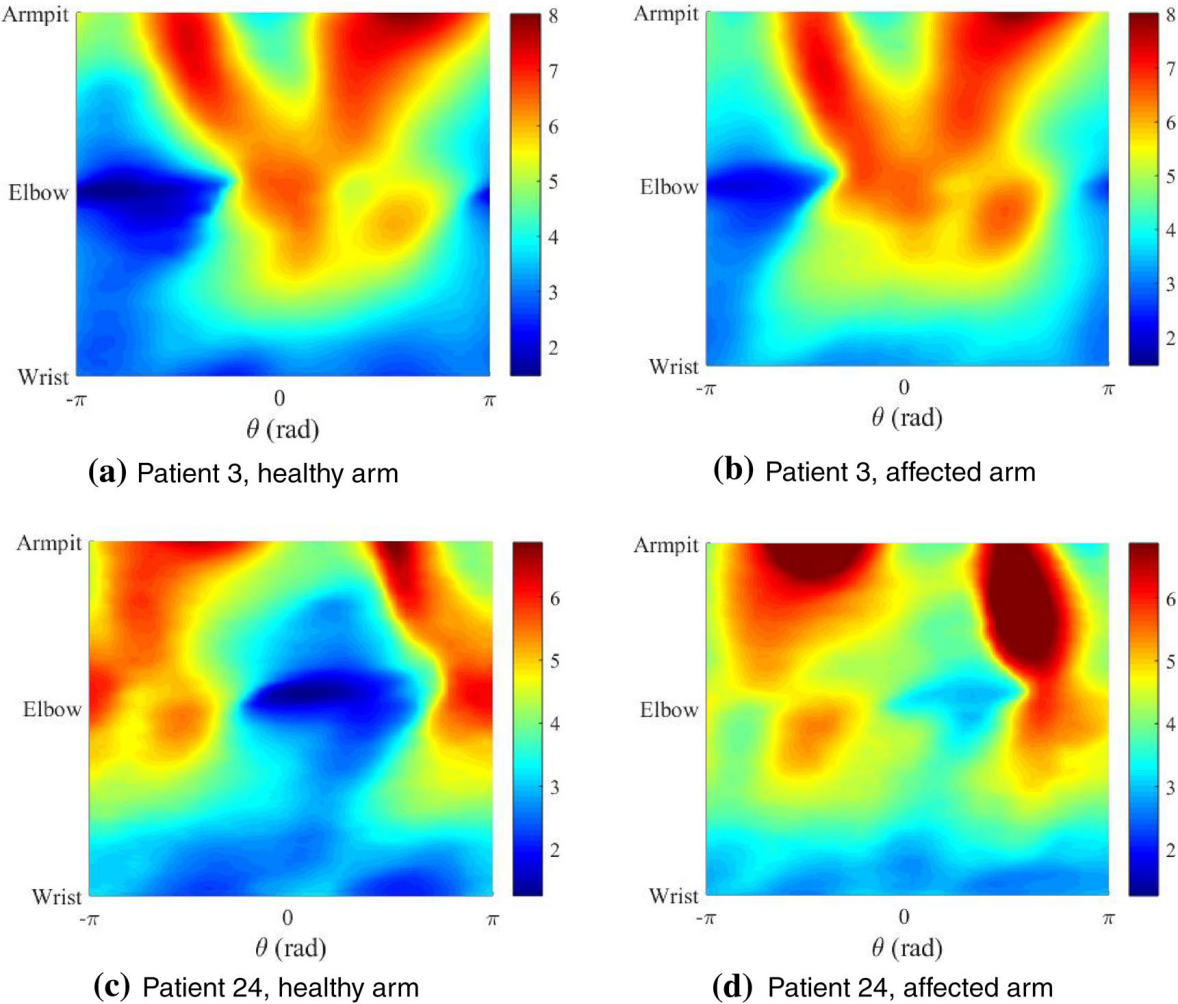
Radial contour maps (in cm) for the healthy (left) and affected (right) arms of two patients. Note that the arms have been mirrored and aligned for easy comparison. Red coloured regions depict larger radial values relative to blue coloured regions.

Figure 7 shows the radial maps for both arms of two patients. There are clear similarities to be recognised between these maps as both arms for a given person are typically similar. As expected, the radial values in Figs. 7b and 7d are overall higher than those in Figs. 7a and 7c, respectively. However, these radial maps are not particularly informative if one is interested in swelling changes. Therefore, a map showing the radial differences can be useful to identify those areas that experience most swelling. This requires three steps in terms of alignment of the graphs. The first step is mirroring of the arm. The second and the third step require a shift in axial and circumferential direction as described in the “Materials and Methods”.

**Figure 8:**
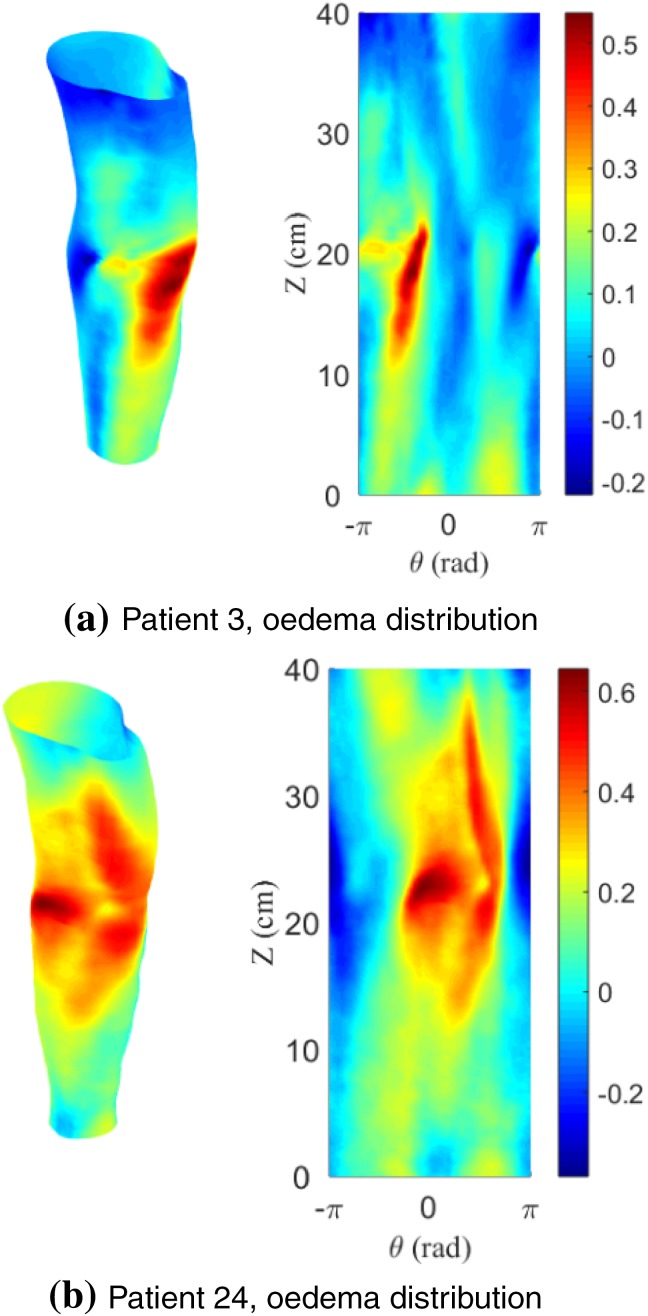
Normalised oedema maps of two patients where the relative radial changes between the healthy and affected arm are projected on the arm and mapped as a $\theta - z$ graph (right). Colour scale increases from negative, areas where healthy arm is larger than affected (blue), to positive (depicting swollen regions in red).

**Figure 9:**
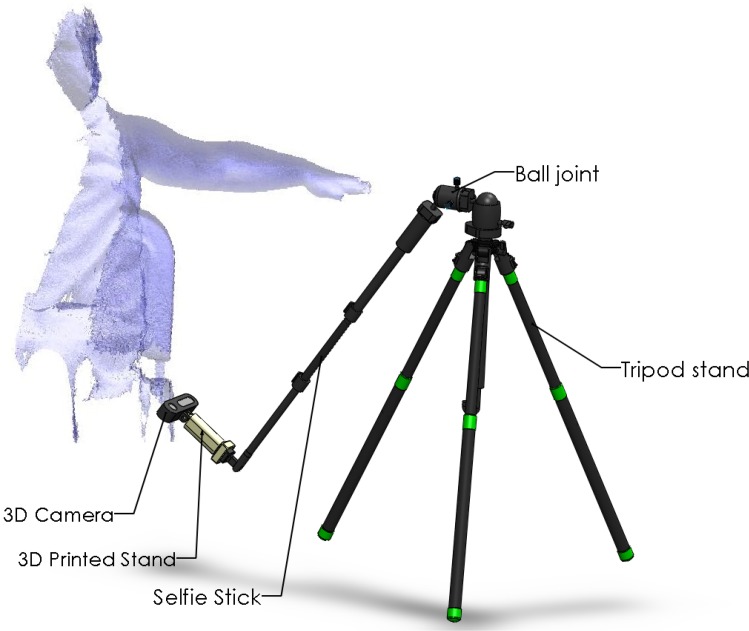
3D drawing of the scanning apparatus. The STL scan, shown in blue, illustrates the subject’s seating position relative to the rig.

**Figure 10:**
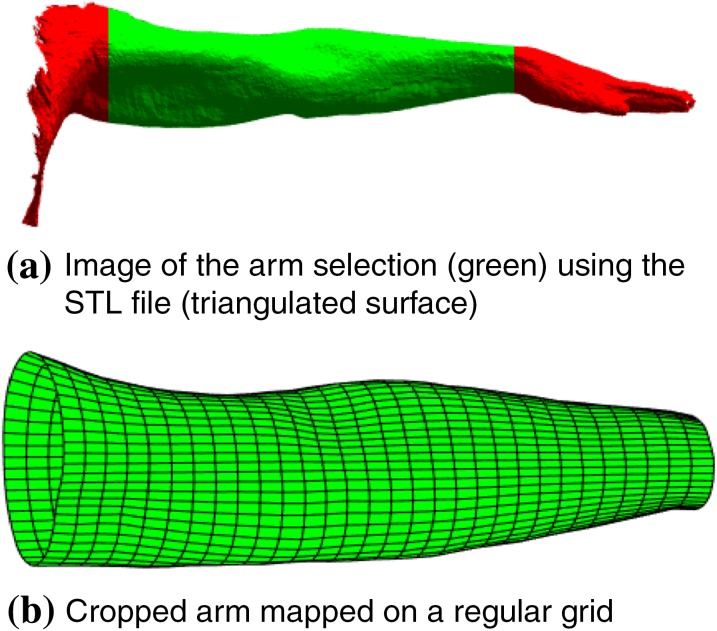
Typical scan after it has been ‘cleaned’ from surrounding clinical environment and unwanted parts from patient’s body.

After subtraction of the radial values for both arms a contour map of the radial changes can be constructed (Fig. 8). A relative measure for swelling was used where the radial difference is scaled with the local radius of the affected arm. The figure clearly shows that the swelling is not homogeneously distributed along the arm, which was already established by looking at circumference and cross-sectional area distributions in the previous section (Fig. 4b). However, the oedema maps identify the areas of swelling in much more detail.

## Discussion

A framework is presented to explore the variability in shape related metrics for lymphoedema patients, alongside the conventional volume measurements. The time taken to scan a subject was roughly 20–30 s, which is considerably faster than the typical 10 min allocated for CLM. Furthermore, the scanning can give instant visual feedback to the specialist. The ICC value, based on circumference and volume calculations (Table 1), falls within the region of good to high reliability, comparable to that of perometry and bioimpedance.^1^^–^ 3,10,25 It can therefore be concluded, along with the coefficient of variance, that the 3D camera is able to repeatedly measure the same amount of volume.

An advantage of having such 3D scans was the ability to refine sampling intervals down to 0.1 cm spacing. This would not be feasible in clinical settings when using tape measurements. Hence, such advantage was utilised to address questions regarding tape measurement spacing and the choice of the geometric formulae to be used. The effect of the widely used, but less accurate frustum and disc methods (assuming the arm to be circular) was quantified through a comparison with the more exact integral method for calculating volume. Using the common 4 cm spacing, both circular methods overestimated by ≈ 100 mL (Fig. 2). Since circles have the largest area to perimeter ratio, it is expected for such methods to overestimate volume. Increasing the sampling points i.e., decreasing segment length, resulted in a minor decrease of volume on average. It can therefore be concluded that a 4 cm spacing is accurate enough to calculate an arm’s volume (< 0.1% change in mean volume across the cohort when comparing 4–0.1 cm spacing), but it is inaccurate to use circular based methods as errors were introduced across locations of least circularity. Although these were in the range of 0.2–2 mL, their cumulative effect across the 0.3 cm spacing (Fig. 3) resulted in an overestimation (≈ 100 ml), which could hide relevant local information for diagnosing lymphoedema, and could lead to false diagnosis.

Any changes in morphology due to oedema would consequently change circularity and circumference of affected arms relative to the healthy arm. This was proved using the paired sample t-test (*p* ≤ 0.05). Both metrics were able to distinguish affected arms from healthy ones. On average, affected arms had larger circumference measurements, and their lower arms became more circular with oedema. The introduction of circularity provided the ability to localise positions across the arm most sensitive to oedema. The wrist and elbow regions were the only positions sensitive to changes using both metrics. Therefore, based on our initial results, clinicians could target those regions to monitor or even detect the incidence of lymphoedema. Early detection and treatment of lymphoedema could restrict irreversible tissue damage.^8^ Furthermore, Soran *et al*. showed a decline in the incidence of breast cancer related lymphoedema due to early and constant monitoring of lymphoedema.^21^ Not only does this benefit the patient’s physical and psychological comfort, it could also reduce unnecessary treatment costs. In another study aiming to create cut-off points for the onset of lymphoedema,^5^ the wrist had the lowest cut-off value when comparing healthy dominant and non-dominant arms, correlating well with our results. Findings from our study related to the elbow could not be compared to theirs, as the authors took 10 cm long segments from the wrist, making it difficult to ascertain the position of the elbow for different patients. Nonetheless, having such pre-determined areas to study could highly reduce CLM procedure times for the detection/monitoring of unilateral lymphoedema.

The circularity metric was also used to distinguish patient groups with Fig. 6 indicating different clusters of patients based on volume and circularity. Such graphs, have the potential to aid the (longitudinal) assessment of patients with similar arm volumes, when coupled with patient outcome. Furthermore, when comparing lower and upper arms, results indicated different groupings of patients. Such tools would be useful to monitor lymphoedema and answer questions such as; which region of the arm is most influenced by the disease? Is treatment more effective in lower/upper arm? Do patients feel better when they fall within a certain region on the graph? However, further investigation is required to establish any correlations with patient outcomes.

The swelling maps (Fig. 8) show that oedema typically occurs around the elbow. This was confirmed by the lymphoedema specialist (third co-author in this work). The maps provide valuable insights on how swelling is distributed along the arm, and can be used to increase treatment efficacy by targeting patient specific needs i.e., the higher resolution of 3D scans vs. CLM results in a detailed visualisation of where treatment should be targeted. We acknowledge the fact that volume would be influenced by arm dominance as discussed by Dylke *et al*.^5^ Healthy dominant arms would have larger circumference measurements, and could lead to the visualisation of false oedemic regions. Further investigation is required to see if shape is also dependent. Nonetheless, it could be hypothesized that circularity would be independent of arm dominance as an increase in circumference, related to dominance, would not necessarily be associated with change in outer shape. In fact, there was no statistical difference, using circularity, between dominant and non-dominant arms of the healthy control group (*p* ≤ 0.05, *N* = 7).

In summary, the tools created were able to separate affected from healthy arms, provide valuable information regarding locations most sensitive to oedema (elbow and wrist), aiding lymphoedema diagnosis/monitoring, create different patient groupings, and easily target swollen regions *via* 3D visualisation of different metrics across the arm. Such data, along with the circularity graphs, could also be used to monitor the longitudinal progression of lymphoedema. The study could also facilitate the creation of new cut-off points for the onset of lymphoedema using topology and shape rather than volume. Furthermore, the ability to visualise treatment efficacy on the arm provides immediate feedback to patients/specialists and could encourage treatment compliance. Future work will focus on longitudinal studies with larger cohorts to investigate if there are any correlations between these metrics and patient outcomes.

## Figures and Tables

**Figure 11:**
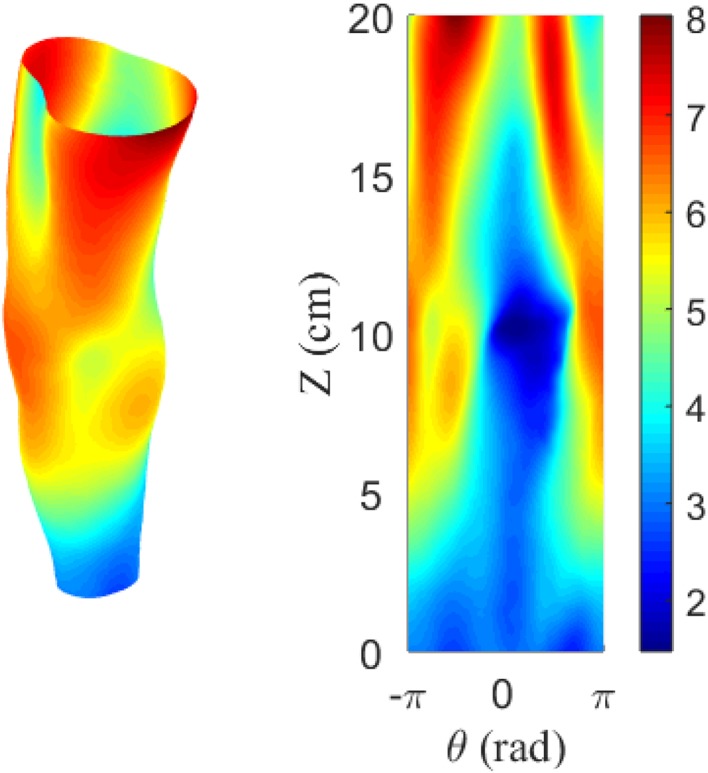
A 2D and 3D visualisation of the radial distribution for an arm. The radial values are in cm.

## Appendix

### Appendix A: Scanning Apparatus

A 3D drawing of the rig used is illustrated below. The subject sits across the selfie stick, with their arm stretched horizontally at the same level as the ball joint. The scanner would hold the selfie stick by the ball joint, and rotate it 360° around the subject’s limb, along with the 3D camera (Fig. 9).

### Appendix B: Pre-processing

1. *Cleaning* Despite the development of a rigorous procedure the outputted STL-files often incorporated unwanted information/artifacts, such as clinical surroundings and undesirable parts of the patients themselves. These artifacts were removed using an algorithm built in the scientific computing software MATLAB. The axial position of an arm within a given scan is typically located at about 70% of the total scan height. The faces of unwanted vertices below and above this location were removed allowing better control on the remaining geometry. To solely attain the arm, a region of interest (ROI) having the shape of a spheroid was created and centred around the geometry’s centre of mass. Initially, this centre of mass sits relatively close to the arm yet is skewed due to the presence of other unwanted features such as the patient’s body and tripod. Therefore, the ROI was made wide enough transversely to incorporate the arm along with the unwanted features. The centre of mass for the geometry was then calculated based on a non-homogeneous distribution of density by assigning more mass to all the vertices inside the spheroid. Subsequently, the ROI was made smaller in an iterative process, shifting the centre of mass towards the arm’s centre point. Solving the eigenvalue problem $\varvec{Iw}_{\varvec{i}} = \lambda_{i} \varvec{w}_{\varvec{i}}$, where $\varvec{I}$ is the moment of inertia tensor, gave the principle axis of the manifold $\varvec{w}_{\varvec{i}} \varvec{ }$ encompassed by the spheroid. Afterwards, vertices lying radially 15 cm away from the principal axis were removed resulting in the hand, arm and some of the shoulder. Note that for all remaining pre- and post-processing steps MATLAB software was used.
2. *Repositioning and Orientation* The triangulated arm manifold is typically not aligned with one of the main Cartesian axes. As this is very convenient for further pre- and post-processing steps, an automated rotation algorithm was created based on the principal axis of the manifold. The main principle axis of an arm always lies in the axial direction. This was used to align the arm with a given Cartesian axis. It should be noted that the principle axis found was compromised when the hand or body were still part of the geometry. To overcome this problem the procedure was iteratively combined with the cropping algorithm described next.
3. *Cropping and Patching* To assess arm lymphoedema, clinical measurements are typically taken from the wrist to the armpit. Currently the user identifies the wrist manually by clicking on it. For consistency between tape measure and STL data, the arm length, which would end just about the armpit, was given as a multiple of 4 cm distances from the wrist by the clinician. Further axial consistency between healthy and affected arms or between follow-up scans of the same arm was achieved using techniques described in the Materials and Methods section. Once both locations of the arm were cropped (Fig. 10), the cross-sectional ends required patching in order to obtain a closed surface for the volume calculations. Nodes of those triangles lying partially outside the cropped edges were shifted axially onto the cutting plane. This then allowed a simple cross-sectional triangulation at the cutting plane ensuring that all normals were defined outward. Note that the cutting planes were defined perpendicular to the principle axis of the manifold.

### Appendix C: Post-processing

1. *Radial/Diameter Mapping* After the three-dimensional model was successfully cropped and reoriented, it was useful to project the unstructured triangulation onto a structured grid. This projection was most easily performed in cylindrical coordinates. Hence, a transformation from Cartesian $\left( {x_{i} ,y_{i} ,z_{i} } \right)$ to cylindrical coordinates $\left( {r_{i} ,\theta_{i} ,z_{i} } \right)$ was performed for each *i*th node (vertex) in the triangulation. A 2D structured grid of $N_{\theta }$ by $N_{z}$ nodes was then defined with coordinates $(\hat{\theta }_{j} ,\hat{z}_{j} )$. Note that ${-}\pi < \hat{\theta }_{j} \le \pi$ and $0 \le \hat{z}_{j} \le L$ where $L$ is the length of the arm with $\hat{z}_{j} = 0$ at the wrist. For every node $(\hat{\theta }_{j} ,\hat{z}_{j} )$ a triangle $T_{j}$ was now found and the barycentric coordinates for the node within $T_{j}$ were saved. This allowed a mapping of the radius (or any other variable) onto the structured grid. It should be mentioned that the grid can be displayed as a 2D radial colourmap, which was useful for comparisons between arms and further analysis. However, it can also be transformed and presented as a 3D object, which is useful for visualisation purposes, e.g., in a clinical setting. Both options are visualised in Fig. 11, where the points that have the highest radius are displayed in red and those of lowest in blue.

A note should be made that different definitions for radius can be used. The more intuitive definition might be to introduce a centre-line that is curved depending on the geometry of the arm. The radius would then be defined as the distance from the centreline to the arm surface measured in normal direction to that centreline. However, this centreline would be susceptible to local shape changes, which makes comparison harder. Hence, in this work we chose to work from the principle axis as determined in the pre-processing section, as this measure is less sensitive to local shape changes of the arm surface.
